# Parental sexual socialization and contraceptive use in young Black women and adolescent girls

**DOI:** 10.1007/s10865-026-00701-2

**Published:** 2026-08-20

**Authors:** Joni A. Brown, Julia R. Steinberg, Patrick A. Wilson, Christine Dunkel Schetter, Jessica D. Gipson

**Affiliations:** 1 Department of Family Science, University of Maryland School of Public Health, College Park, MD 20742 USA; 2 Department of Psychology, University of California, Los Angeles, CA USA; 3 Department of Community Health Sciences, UCLA Fielding School of Public Health, Los Angeles, CA USA; 4 UCLA Bixby Center to Advance Sexual and Reproductive Health Equity, UCLA Fielding School of Public Health, Los Angeles, CA USA

**Keywords:** Sexual socialization, Contraceptive use, Black women, Adolescent girls, Sexual health, Reproductive health

## Abstract

Contraceptive use is particularly low among young Black women and adolescent girls in the United States. Sociocultural factors such as parent-child communication about sex (i.e., parental sexual socialization) may be relevant to contraceptive use in young Black women and girls. However, no research has identified patterns in the content of sexual socialization messages received by young Black women and adolescent girls in the U.S., nor examined the associations between sexual socialization patterns and contraceptive use in this population. Thus, we performed a latent class analysis using data from the 2013–2019 National Survey of Family Growth from Black women and girls aged 15–24 (*N*_unweighted_ = 794) to identify sexual socialization patterns. We identified four patterns of parental sexual socialization: (1) *Comprehensive*; (2) *Limited*; (3) *Abstinence-focused*; (4) *Contraception-focused*. Binomial and multinomial logistic regression analyses were then performed to investigate the associations between these socialization patterns and contraceptive practices at the first and most recent sexual intercourse. Compared to respondents in the *Contraception-focused* class, those in the *Limited* and *Abstinence-focused* classes were less likely to engage in preventive contraceptive behaviors at the first intercourse. However, there were few differences between the patterns in contraceptive behaviors at the most recent intercourse. These results underscore differences in sexual socialization experiences of young Black women and adolescent girls. The findings also have important implications for identifying those who may be at risk of unintended pregnancies, and for informing Black parents about what messages are most beneficial to promote contraceptive use in Black girls.

## Introduction

Black women in the United States experience high rates of unintended pregnancy (Finer & Zolna, 2016) and short birth spacing (Gemmill & Lindberg, 2013), and Black adolescent girls experience a higher rate of teenage births relative to the national teenage birth rate (Osterman et al., 2022). Furthermore, Black women and adolescent girls report lower use of any contraception, and lower use of highly or moderately effective contraceptive methods, as compared to White women and girls (Dehlendorf et al., 2014). Contraception plays an essential role in managing one’s fertility, particularly as it relates to achieving one’s desired number of children and the timing of pregnancies. However, there are various factors that influence people’s contraceptive behaviors, including individual-, interpersonal-, and institutional-level factors (Dehlendorf et al., 2010). One interpersonal-level factor that may be particularly relevant to the sexual and reproductive well-being of Black women and adolescent girls is parental sexual socialization.

### Parental sexual socialization and contraceptive use

Parental sexual socialization describes the process whereby children receive direct and indirect messages about sex and other related topics from their parents or caregivers to help shape their attitudes and behaviors (Shtarkshall et al., 2007). Socialization practices often reflect families’ social and cultural values and beliefs around sex. For Black families, in particular, the sexual socialization messages they relay to Black girls may be influenced by their intersectional status in society (i.e., being both Black *and* female) (Crenshaw, 1989). Thus, families may relay sexual socialization messages that reflect their concerns about how Black girls may be viewed in society due to harmful stereotypes of Black women’s sexuality (e.g., Jezebel stereotype which characterizes Black women as promiscuous and sexually aggressive) (Leath & Mims, 2021). Research shows that Black families in the U.S. may convey a variety of messages about sex to Black girls, including instructive messages such as educational messages about contraception, and messages about risks of engaging in sex (e.g., pregnancy, sexually transmitted infections) (Brown et al., 2024; Dennis & Wood, 2012; Fasula et al., 2007; Frederick et al., 2024; Nwoga, 2000), though some may not talk about sex at all (Crooks et al., 2019; Evans & Dyson, 2015; Leath et al., 2020; Logan et al., 2021).

Parental sexual socialization may play an important role in promoting safe sexual practices that reduce risk of pregnancy and sexually transmitted infections. In particular, sexual socialization messages may help to guide attitudes around contraception and contraceptive practices in young people (Coakley et al., 2017). Indeed, a meta-analysis of 52 studies found a small but significant positive association between parent-adolescent communication about sex and contraceptive use in adolescents and young adults (Widman et al., 2016). Furthermore, moderation analyses indicated that this association was significantly stronger among girls than boys. Only a few studies have examined the association between parental sexual socialization and contraceptive practices exclusively in young Black women and adolescent girls, and the results are inconsistent. Some research on Black adolescent girls and college women has found that less frequent communication with parents is associated with not using condoms or any form of contraception during sexual intercourse (DiClemente et al., 2001; Hutchinson & Montgomery, 2007). However, another study found no association between mother-daughter communication about sex and condom use in sexually active Black girls (Donenberg et al., 2011).

### Patterns of parental sexual socialization and sexual behaviors

Quantitative studies of parental sexual socialization often employ measures that assess the *frequency* of parent-child communication (Bynum, 2007) or the *number* of sex-related topics discussed (DiIorio et al., 1999). However, these methods of assessing parental sexual socialization do not account for patterns of socialization messages (i.e., the content of the sexual socialization messages) and how they may differ between families. For example, two study participants may report a similar *frequency* of sex communication with their parents or the same *number* of sex-related topics discussed, but the topics of the sex-related communications may be different from each other. That is, one participant may receive messages around abstaining until marriage, while another may receive messages around using contraception if engaging in sex. Indeed, parents may tailor their socialization messages based on various factors including their perceived knowledge about specific topics, the gender of their children, and their children’s level of sexual activity (Beckett et al., 2010; DiIorio et al., 2003). Furthermore, children may receive differing and even contradictory socialization messaging from their mothers and fathers, which may contribute to their unique sexual socialization experiences (DiIorio et al., 1999). Thus, methodological approaches which consider patterns of sexual communication based on the topics or content (also known as person-centered approaches) may better capture differences in the parental sexual socialization experiences of adolescents and young people (Lanza & Cooper, 2016).

Only one study to date has employed a person-centered analytic technique to assess patterns of parental sexual socialization content (Mutumba et al., 2024). In a large sample of adolescent girls in Southwestern Uganda, the researchers used latent class analyses to identify three patterns of parental sexual socialization based on their experiences of receiving messages around six topics: HIV/AIDS, sexually transmitted diseases (STDs), sex, puberty, pregnancy, and marriage. The first class (*Avoidant*) was characterized by high probabilities of receiving messages about puberty and pregnancy, and low to moderate probabilities of receiving messages about marriage, HIV, STDs and sex. The second class (*Functional*) was characterized by high probabilities of receiving messages about HIV, STDs, puberty and pregnancy, and low to moderate probability of receiving messages about sex and marriage. The third and final class (*Comprehensive*) was characterized by moderate to high probabilities of receiving messages about all six topics. In addition to identifying these socialization patterns, the researchers examined associations between the patterns and adolescent girls’ sexual risk-taking attitudes, but there were no significant associations. However, this study did not address whether the socialization patterns are associated with sexual behaviors, particularly contraceptive use.

### The current study

There is a dearth of research assessing patterns in the content of parental sexual socialization, and no study to date has identified patterns of parental sexual socialization, nor potential associations between patterns of parental sexual socialization and contraceptive use among young Black women and girls in the United States. The few studies published thus far on parental sexual socialization and contraceptive use have focused primarily on condom use, with little attention given to other contraceptive methods. Furthermore, to our knowledge, no study has examined the relation between parental sexual socialization and the number of contraceptive methods that young Black women and girls use during sexual intercourse, despite research indicating that concurrent use of multiple methods is increasing among women of reproductive age (Kavanaugh et al., 2021). Therefore, the aims of the present study were (1) to identify patterns of parental sexual socialization messages as reported by young Black women and girls, (2) to describe how these patterns differ by sociodemographic characteristics, age at first sexual intercourse, and number of socialization messages received, and (3) to examine the associations between the patterns and contraceptive practices at the first and most recent acts of sexual intercourse.

## Method

### Study design and sample

The current study used data from the National Survey of Family Growth (NSFG) collected between 2013 and 2019. The NSFG is a cross-sectional, nationally representative survey of all non-institutionalized women and men aged 15–49 in the United States that gathers information on sexual and reproductive health. The survey uses a stratified, multi-stage area probability sampling design and oversamples Hispanic persons, non-Hispanic Black persons, and adolescents aged 15–19. Details on the sampling design and survey procedures of the NSFG are available elsewhere (National Center for Health Statistics, 2009).

NSFG data from the 2013–2015, 2015–2017 and 2017–2019 public-use data files were combined. Since the analyses for this study focused on contraceptive use, the analytic sample was restricted to U.S.-born, non-Hispanic Black female respondents aged 15–24 who reported ever having heterosexual vaginal intercourse and who did not report sterility at the time of their first intercourse. We specified this age range because questions on parental sexual socialization were administered only to respondents aged 15–24. Respondents who met the specified criteria but who did not have data on their parental sexual socialization experiences were excluded (*n*_unweighted_ = 3). This resulted in an unweighted sample of 794 Black female respondents. This study did not meet the criteria for human subject research, and was therefore exempt from review by the University of Maryland Institutional Review Board.

## Measures

### Sexual socialization

Sexual socialization was measured with the following question: “(Before you were 18 years old, ) which, if any, of the topics shown on Card 23 (did you ever talk/have you ever talked) with a parent or guardian about?” The topics included: (1) how to say no to sex, (2) methods of birth control, (3) where to get birth control, (4) sexually transmitted diseases, (5) how to prevent HIV/AIDS, (6) how to use a condom, (7) waiting until marriage to have sex. Binary variables were created for all seven topics to indicate whether respondents received a specific message (“yes” or “no”). These seven items were used as indicators in a latent class analysis to identify patterns of sexual socialization (see Data Analysis and Results sections and Supplementary Material for more information on the patterns). In addition, we created a composite score of sexual socialization for descriptive purposes by summing the seven items.

### Contraceptive use at first sexual intercourse

Three variables were created to assess contraceptive use at the first sexual intercourse using recoded variables by the NSFG on the types of contraceptive methods used at the first intercourse: (1) use of any contraception; (2) most effective type of contraceptive method used; (3) number of contraceptive methods used. *Use of any contraception* was a binary variable to indicate whether any form of contraception was used at the first intercourse (“yes” or “no”). *Most effective type of contraceptive method used* was a categorical variable used to classify respondents according to the different types of contraception as described by The Guttmacher Institute (2021), which include long-acting reversible methods (i.e., intrauterine device, implant), short-acting reversible methods (i.e., pills, patch, vaginal ring, injection), coital methods (i.e., condom, diaphragm, cervical cap, sponge, spermicide or vaginal gel, fertility awareness-based methods, withdrawal method). This variable was constructed as such to account for respondents who reported use of more than one contraceptive method at the first intercourse. Given that only a small percentage of the sample (2%) reported using long-acting reversible methods at the first intercourse, respondents were therefore classified into one of three categories: (1) long- or short-acting reversible method; (2) coital method; (3) no method. *Number of contraceptive methods used* was a categorical variable to classify respondents into one of three categories: (1) multiple methods used; (2) single method used; (3) no method.

### Contraceptive use at most recent sexual intercourse

Three variables were created to assess contraceptive use at the most recent intercourse using recoded NSFG variables on the types of contraceptive methods used at the last intercourse in the past 12 months: (1) use of any contraception; (2) most effective type of contraceptive method used; (3) number of contraceptive methods used. *Use of any contraception* was a binary variable to indicate whether any form of contraception was used at the last intercourse in the past 12 months. *Most effective type of contraceptive method used* classified respondents into one of four categories according to the most effective method used at the last intercourse: (1) long-acting reversible method; (2) short-acting reversible method; (3) coital method; (3) no method. Another version of this variable was created to use in sensitivity analyses by combining long- and short-acting reversible methods. *Number of contraceptive methods used* classified respondents into one of three categories: (1) multiple methods used; (2) single method used; (3) no method.

### Covariates

The selection of covariates was informed by previous research on correlates of contraceptive use in young women (Frost et al., 2007; Manning et al., 2000). Sociodemographic covariates included poverty level, family structure when growing up, religious affiliation, and relationship with male sexual partner at the time of the first and most recent sexual encounters. Because the NSFG does not directly assess relationship with last sexual partner for female respondents, we created a variable for this construct using available data on respondents’ relationship history with their male sexual partners based on the method outlined by Copen (2017). Specifically, we utilized respondent’s data on the relationship at first sex with their first sexual partner, and if applicable, data on their current relationship with their first partner, relationship at last sex with first sexual partner, relationship at first sex with their last partner ever, current relationship with their last partner ever, and relationship at last sex with last partner ever. Thus, respondent’s relationship status at the most recent intercourse was classified according to available data on their relationship with their most recent male sexual partner. In addition to those covariates, age at first intercourse was included in multivariable models concerning contraceptive use at the first intercourse, whereas age at interview, duration of health insurance coverage in the past year and parity were included in models concerning contraceptive use at the most recent intercourse.

## Data analysis

A latent class analysis (LCA) was performed to identify patterns in the reports of sexual socialization messages received. LCA is a person-centered approach that identifies underlying groups in a population that are qualitatively different, using observed categorical indicators (Nylund-Gibson & Choi, 2018; Weller et al., 2020). Class enumeration was performed to determine the class solution that best fits the data. The model solutions were then compared on the following information criteria: (1) Bayesian Information Criterion (BIC); (2) Sample-size adjusted Bayesian Information Criterion (SABIC); (3) Akaike Information Criterion (AIC); (4) Consistent Akaike Information Criterion (CAIC). For these fit indices, lower values indicate better fit for the data (Nylund-Gibson & Choi, 2018).

Diagnostic criteria information were used to assess how well each model solution differentiates between classes. The Vuong-Lo-Mendell-Rubin Likelihood Ratio Test (VLMR-LRT) compares model fit improvement between a model and the preceding model, such that *p* values less than .05 indicate improved fit from the preceding model. Average posterior probabilities (AvePP) provide information on the accuracy of the models in predicting individuals’ class memberships, with values greater than 0.70 indicating greater accuracy in predicting class membership. Entropy provides information on how well a model separates between its classes, with values greater than 0.80 indicating acceptable class separation. Item probability plots were also inspected to determine the best fit model.

After the best fit model was identified, bivariate analyses were performed to characterize the socialization classes by examining class differences in the sociodemographic covariates, age at first sexual intercourse, and the total number of sexual socialization messages received. To examine whether the latent classes were associated with contraceptive use at the first and most recent acts of intercourse, a series of unadjusted and adjusted binomial and multinomial logistic regression models were performed using dummy coded variables of class membership. For the multinomial analyses concerning the most effective type of method used, we compared “long- or short-acting reversible method” to all other categories for the first sex, and “long-acting reversible method” to the other categories for the most recent sex. In addition, we performed a sensitivity analysis to compare “long- or short-acting reversible method” to the other categories at the most recent sex, to replicate the analysis of the most effective type of method used at first sex. For the analyses on the number of methods used at both acts of intercourse, “multiple methods” was compared to “single method” and “no method.”

The full sample (*N*_unweighted_ = 794) was used for the analyses predicting contraceptive use at the first intercourse. For the analyses predicting contraceptive use at the most recent intercourse, we used a subsample of respondents who reported having had sex more than once, having had sex in the past 12 months, and who were at risk of having an unintended pregnancy (i.e., not pregnant or trying to become pregnant, and no report of sterility) (*N*_unweighted_ = 596). These criteria were specified so as to distinguish the most recent act of intercourse from the first act of intercourse. Descriptive and bivariate analyses were performed in R (R Core Team, 2022) and RStudio (RStudio Team, 2023) using the “survey” package (Lumley, 2004) to account for the complex survey design. The LCA and regression analyses were performed in Mplus (Version 8.9) (Muthén & Muthén, 2017) to account for the complex survey design and to use full information maximum likelihood estimation to address the missing data (Enders, 2001).

## Results

### Sample characteristics and descriptive results

See Table 1 for the sample characteristics. The mean age of respondents at the time of the interview was 21 years (*M* = 20.58, *SD* = 2.54). Regarding socioeconomic status, 77% of respondents reported having a high school diploma or higher education, and 43% reported a family income below 100% of the poverty level. A large majority of respondents (86%) resided in a metropolitan area. Approximately one-quarter of the sample (27%) reported living with two biological or adoptive parents during their childhood, and just over three-quarters of the sample (76%) reported having a religious affiliation. Specifically, 69% of respondents reported being Protestants, while 4% reported being Catholic and 3% reported other religious affiliations.

**Table 1:** Sociodemographic characteristics across NSFG surveys in 2013–2015, 2015–2017 and 2017–2019

Characteristics	Full sample (*N* = 794)	2013–2015 (*N* = 272)	2015–2017(*N* = 285)	2017–2019 (*N* = 237)
Age at interview	20.58 (2.54)	20.54 (2.52)	20.68 (2.54)	20.50 (2.57)
Age at first sexual intercourse since menarche	16.09 (1.84)	15.92 (2.00)	16.07 (1.85)	16.30 (1.61)
*Educational attainment*				
Less than high school	211 (24)	73 (27)	75 (22)	63 (23)
High school graduate	321 (37)	103 (36)	119 (42)	99 (32)
Some college but no degree	194 (29)	76 (29)	67 (25)	51 (34)
Associate degree	28 (4)	7 (1)	11 (6)	10 (3)
Bachelor’s degree or higher	40 (7)	13 (7)	13 (5)	14 (8)
*Poverty level*				
Less than 100% of poverty level	398 (43)	149 (51)	153 (47)	96 (31)
100–200% of poverty level	196 (29)	58 (26)	66 (28)	72 (34)
More than 200% of poverty level	200 (28)	65 (24)	66 (25)	69 (35)
*Past-year duration of health insurance coverage*				
Full-year coverage	605 (77)	202 (78)	218 (75)	185 (78)
Partial-year coverage	86 (12)	38 (13)	28 (11)	20 (13)
No coverage	81 (11)	27 (9)	28 (15)	26 (10)
*Place of residence*				
Principal city of a metropolitan statistical area (MSA)	392 (47)	127 (45)	140 (41)	125 (57)
Other MSA	265 (39)	88 (41)	92 (41)	85 (35)
Non-MSA	137 (14)	57 (14)	53 (19)	27 (8)
*Childhood family structure*				
Two biological or adoptive parents from birth	204 (27)	60 (25)	83 (29)	61 (27)
Other family composition	590 (73)	212 (75)	202 (71)	176 (73)
*Religious affiliation*				
Affiliated with a religion	640 (76)	217 (77)	235 (78)	188 (71)
No religious affiliation	154 (24)	55 (23)	50 (22)	49 (29)
*Religion*				
Protestant	575 (69)	195 (70)	208 (69)	172 (67)
Catholic	33 (4)	14 (5)	13 (5)	6 (3)
Other religion	32 (3)	8 (2)	14 (4)	10 (2)
None	154 (24)	55 (23)	50 (22)	49 (29)

Data are presented as unweighted frequencies and weighted percentages for categorical variables, and weighted means and standard deviations for continuous variables

Approximately one-half of respondents reported receiving each of the seven socialization messages (see Table 2 for details). Regarding contraceptive use at the first sexual intercourse, nearly three-quarters of respondents (72%) reported using some form of contraception, with 19% of respondents reporting a long- or short-acting reversible method as the most effective type of contraception used. Approximately 15% of respondents reported using multiple methods at first sex, of which almost all (99.8%) reported a condom as one of their chosen methods. Related to contraceptive use at the most recent intercourse, most respondents (80%) reported using some form of contraception, with 13% of respondents reporting a long-acting reversible method as the most effective method used. About 26% of respondents reported using multiple methods at the most recent sex, of which 77% reported using a condom as well as another method.

**Table 2:** Descriptive statistics on study variables (N = 794)

Variable	*N* (%)
*Sexual socialization messages * ^a^	
How to say no to sex	464 (58)
Methods of birth control	467 (57)
Where to get birth control	393 (49)
Sexually transmitted diseases	483 (57)
How to prevent HIV/AIDS	393 (47)
How to use a condom	389 (47)
Waiting until marriage to have sex	459 (59)
*Contraceptive use at first sexual intercourse*	
Yes	561 (72)
No	233 (28)
*Most effective type of contraceptive method used at first sexual intercourse*	
Long- or short-acting reversible method	133 (19)
Coital method	428 (53)
No method	233 (28)
*Number of contraceptive methods used at first sexual intercourse*	
Multiple methods used	107 (15)
Single method used	454 (57)
No method	233 (28)
*Contraceptive use at most recent sexual intercourse * ^b^	
Yes	506 (80)
No	90 (20)
*Most effective type of contraceptive method used at most recent sexual intercourse * ^b^	
Long-acting reversible method	71 (13)
Short-acting reversible method	180 (28)
Coital method	255 (39)
No method	90 (20)
*Number of contraceptive methods used at most recent sexual intercourse* ^b^	
Multiple methods used	132 (26)
Single method used	374 (55)
No method	90 (20)

Data are presented as unweighted frequencies and weighted percentages

^a^Frequencies and percentages reflect respondents who reported receiving each item

^b^Only respondents who reported having sex in the past 12 months and who reported having sex more than once (*N*_unweighted_ = 596)

### Latent class analysis

**Fig. 1:**
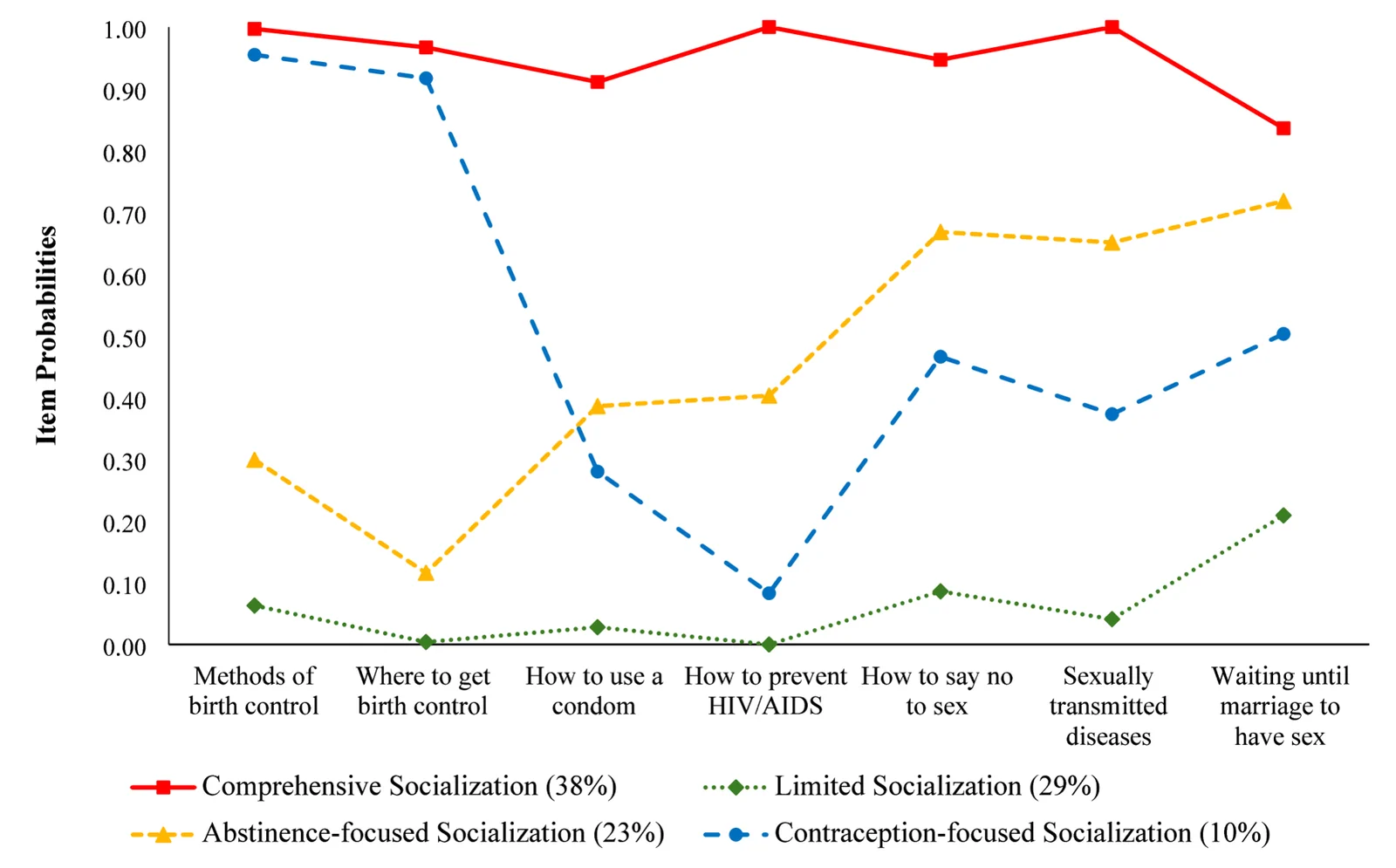
Probability of receiving sexual socialization messages by patterns of parental sexual socialization (N_unweighted_ = 794)

Six latent class models were estimated using the seven sexual socialization items. Fit statistics for these models are presented in Supplementary Table 1. The superior fit indices were concentrated around the 4-, 5-, and 6-class models. In particular, the 4-class model had the lowest BIC, the 5-class model had the lowest SABIC and CAIC, and the 6-class model had the lowest AIC. However, there was very little difference in CAIC values between the 4- and 5-class models. The VLMR-LRT indicated that the 2-class model improved the model fit over the 1-class model, and that the 3-class model showed improvement over the 2-class model. Furthermore, all six models had acceptable values for entropy and AvePP. Given that there was strong statistical support for either the 4- or 5-class model, we inspected the item probability plots for these class solutions (see Fig. 1 and Supplementary Fig. 1) and determined that the 4-class model had more interpretable classes than the 5-class model. Thus, the 4-class model was identified as the best fit model.

Figure 1 displays the item probability plot for the 4-class model. The largest class was labeled *Comprehensive Socialization*, with 38% of the sample classified in this group. This class was characterized by high probabilities of receiving all seven sexual socialization messages. The second largest class was labeled *Limited Socialization* (29% of the sample). In contrast to the *Comprehensive Socialization* class, this class was characterized by low probabilities of receiving all seven sexual socialization messages. The third largest class was labeled *Abstinence-focused Socialization* (23% of the sample). This class was characterized by high probabilities of receiving messages such as “how to say no to sex,” “waiting until marriage to have sex,” and “sexually transmitted diseases,” and low to moderate probability of receiving all other messages which mostly concerned contraception. The final and smallest class was labeled *Contraception-focused Socialization* and had 10% of the sample. This class was characterized by high probabilities of receiving contraception-specific messages such as “methods of birth control” and “where to get birth control,” but a low probability of receiving messages about how to use a condom. Given that the *Contraception-focused Socialization* class was characterized by having received messages that primarily centered contraception, this class was used as the reference group in the analyses examining the associations between the patterns of socialization and contraceptive use.

### Differences between parental sexual socialization patterns

Findings regarding whether and how sociodemographic characteristics, age at first sexual intercourse, and number of sexual socialization messages received vary by the sexual socialization patterns are presented in Supplementary Table 2. There was a statistically significant association between the socialization patterns and religious affiliation. Among respondents who reported having a religious affiliation, only 7% were classified in the *Contraception-focused Socialization* class, whereas 18% of respondents without a religious affiliation were classified as such. In addition, 25% of religiously affiliated respondents were classified in the *Abstinence-focused Socialization* class compared to 15% of respondents without a religious affiliation. There were no significant associations between the patterns and the other sociodemographic factors. In addition, there was a significant association between the patterns and age at first intercourse, such that respondents in the *Limited Socialization* class were significantly younger at the first intercourse (*M* = 15.44, *SE* = 0.18) compared to those in the *Comprehensive Socialization* class (*M* = 16.31, *SE* = 0.15) and the *Abstinence-focused Socialization* class (*M* = 16.53, *SE* = 0.20).

We also compared the mean number of sexual socialization messages received by the four socialization patterns (see Supplementary Table 2). Respondents in the *Comprehensive Socialization* class received significantly more socialization messages (*M* = 6.65, *SE* = 0.06) than those in the *Limited Socialization* (*M* = 0.37, *SE* = 0.05), *Abstinence-focused Socialization* (*M* = 3.23, *SE* = 0.09), and *Contraception-focused* (*M* = 3.66, *SE* = 0.15) classes. In addition, respondents in the *Limited Socialization* class received significantly less messages than those in the *Abstinence-focused* and *Contraception-focused* classes. On average, respondents in the *Abstinence-focused* and *Contraception-focused* classes received the same number of socialization messages.

### Associations between sexual socialization patterns and contraceptive practices

#### Use of any contraception

Table 3 presents the unadjusted and adjusted results from the binomial logistic regression analyses examining whether the patterns of sexual socialization are associated with use of any form of contraception. Compared to respondents in the *Contraception-focused Socialization* class, those in the *Limited Socialization* (adjusted Odds Ratio [aOR] = 0.33, 95% CI 0.13–0.83) and *Abstinence-focused Socialization* (aOR = 0.36, 95% CI 0.16–0.83) classes were less likely to have used contraception at first sex, even after adjusting for covariates. Similarly, compared to respondents in the *Contraception-focused* class, those in the *Limited Socialization* class were less likely to have used contraception at the most recent sex (OR = 0.29, 95% CI 0.09–0.92). However, this was not significant after adjusting for covariates (aOR = 0.33, 95% CI 0.10–1.06, *p* = 0.06). There were no significant differences in contraceptive use at the first or most recent sex between those in the *Comprehensive Socialization* and *Contraception-focused* classes.

**Table 3:** Associations between sexual socialization patterns and use of any contraception

Sexual socialization patterns	First sexual intercourse (*N*= 794)		Most recent intercourse (*N*= 596)	
	OR (95% CI)	aOR (95% CI)	OR (95% CI)	aOR (95% CI)
*Contraception-focused socialization (Ref)*				
Comprehensive socialization	0.54 (0.24–1.25)	0.55 (0.24–1.26)	0.36 (0.11–1.17)	0.40 (0.14–1.19)
Limited socialization	**0.30 (0.12–0.73)**	**0.33 (0.13–0.83)**	**0.29 (0.09–0.92)**	0.33 (0.10–1.06)
Abstinence-focused socialization	**0.36 (0.16–0.81)**	**0.36 (0.16–0.83)**	0.58 (0.17–1.96)	0.69 (0.22–2.17)

Ref , Reference group; OR , Odds ratio; aOR , Adjusted odds ratio. Statistically significant findings are bolded. For contraceptive use at first sexual intercourse, adjusted models controlled for age at first sex, poverty level, childhood family structure, religious affiliation, and relationship with first male sexual partner. For contraceptive use at most recent intercourse, adjusted models controlled for age at interview, poverty level, childhood family structure, religious affiliation, relationship with last male sexual partner, duration of past-year health insurance coverage, and parity

### Most effective type of contraceptive method used

Table 4 presents the unadjusted and adjusted results from the multinomial logistic regression analyses examining whether the socialization patterns are associated with the most effective type of contraceptive method used. Compared to respondents in the *Contraception-focused* class, those in the *Abstinence-focused* class were less likely to have used a long- or short-acting reversible method at first sex than a coital method (aOR = 0.23, 95% CI 0.07–0.74) or no method at all (aOR = 0.12, 95% CI 0.04–0.35). Similarly, compared to respondents in the *Contraception-focused* class, those in the *Limited Socialization* class were less likely to have used a long- or short-acting reversible method at first sex than no method at all (aOR = 0.20, 95% CI 0.06–0.65). There was no significant difference in the most effective type of method used at first sex between respondents in the *Comprehensive* and *Contraception-focused* classes. Likewise, there were no significant differences between the socialization patterns in the most effective type of method used at the most recent intercourse. Sensitivity analyses (see Supplementary Table 3) indicated that compared to respondents in the *Contraception-focused* class, those in the *Limited Socialization* class were less likely to have used a long- or short-acting reversible method than no method at the most recent sex in the unadjusted model (OR = 0.25, 95% CI 0.07–0.94), but not in the adjusted model (aOR = 0.29, 95% CI 0.08–1.03, *p* = 0.06).

**Table 4:** Associations between sexual socialization patterns and the most effective type of contraceptive method used

	Most effective method used at first sexual intercourse (*N*= 794)				Most effective method used at most recent intercourse (*N*= 596)					
	LSARC versus no method		LSARC versus coital method		LARC versus no method		LARC versus coital method		LARC versus SARC	
Sexual socialization patterns	OR (95% CI)	aOR (95% CI)	OR (95% CI)	aOR (95% CI)	OR (95% CI)	aOR (95% CI)	OR (95% CI)	aOR (95% CI)	OR (95% CI)	aOR (95% CI)
*Contraception-focused socialization (Ref)*										
Comprehensive socialization	0.42 (0.13–1.34)	0.41 (0.14–1.20)	0.66 (0.22–1.95)	0.64 (0.23–1.79)	0.24 (0.03–1.75)	0.29 (0.06–1.54)	0.53 (0.10–2.93)	0.61 (0.14–2.57)	0.71 (0.12–4.14)	0.72 (0.16–3.16)
Limited socialization	**0.17 (0.04–0.65)**	**0.20 (0.06–0.65)**	0.44 (0.13–1.47)	0.47 (0.16–1.37)	0.21 (0.03–1.42)	0.22 (0.04–1.14)	0.59 (0.11–3.34)	0.55 (0.12–2.47)	0.75 (0.12–4.75)	0.65 (0.14–3.03)
Abstinence-focused socialization	**0.13 (0.04–0.40)**	**0.12 (0.04–0.35)**	**0.25 (0.08–0.81)**	**0.23 (0.07–0.74)**	0.33 (0.05–2.38)	0.38 (0.07–2.05)	0.39 (0.07–2.26)	0.41 (0.09–1.90)	0.67 (0.11–4.27)	0.62 (0.12–3.15)

Ref ,  Reference group; OR , Odds ratio; aOR , Adjusted odds ratio; LSARC, Long- or short-acting reversible contraceptive method; LARC, Long-acting reversible contraceptive method; SARC, Short-acting reversible method. Statistically significant findings are bolded. For contraceptive use at first sexual intercourse, adjusted models controlled for age at first sex, poverty level, childhood family structure, religious affiliation, and relationship with first male sexual partner. For contraceptive use at most recent intercourse, adjusted models controlled for age at interview, poverty level, childhood family structure, religious affiliation, relationship with last male sexual partner, duration of past-year health insurance coverage, and parity

### Number of contraceptive methods used

Table 5 presents the unadjusted and adjusted results from the multinomial logistic regression analyses examining the associations between the sexual socialization patterns and the number of contraceptive methods used. Compared to respondents in the *Contraception-focused Socialization* class, those in the *Limited Socialization* class were less likely to have used multiple methods at first sex than a single method (aOR = 0.23, 95% CI 0.08–0.70) or no method (aOR = 0.10, 95% CI 0.03–0.35). Similar results were found when comparing the *Abstinence-focused* class to the *Contraception-focused* class (No method: aOR = 0.13, 95% CI 0.04–0.43; Single method: aOR = 0.27, 95% CI 0.08–0.91). Likewise, compared to respondents in the *Contraception-focused* class, those in the *Limited Socialization* class were less likely to have used multiple methods versus no method at the most recent sex (aOR = 0.24, 95% CI 0.06–0.98). There were no other significant differences in the number of methods used at the most recent sex between respondents in the *Contraception-focused Socialization* class and those in the other three classes.

**Table 5:** Associations between sexual socialization patterns and number of contraceptive methods used

	Number of methods used at first sexual intercourse (*N*= 794)				Number of methods used at most recent intercourse (*N*= 596)			
	Multiple versus none		Multiple versus single		Multiple versus none		Multiple versus single	
Sexual socialization patterns	OR (95% CI)	aOR (95% CI)	OR (95% CI)	aOR (95% CI)	OR (95% CI)	aOR (95% CI)	OR (95% CI)	aOR (95% CI)
*Contraception-focused socialization (Ref)*								
Comprehensive socialization	0.41 (0.12–1.39)	0.45 (0.15–1.31)	0.65 (0.21-2.00)	0.73 (0.27–1.98)	0.26 (0.06–1.17)	0.29 (0.08–1.10)	0.60 (0.19–1.96)	0.61 (0.20–1.89)
Limited socialization	**0.08 (0.02–0.29)**	**0.10 (0.03–0.35)**	**0.18 (0.05–0.65)**	**0.23 (0.08–0.70)**	**0.20 (0.04–0.91)**	**0.24 (0.06–0.98)**	0.55 (0.15–2.02)	0.60 (0.18–2.03)
Abstinence-focused socialization	**0.13 (0.04–0.44)**	**0.13 (0.04–0.43)**	**0.25 (0.07–0.89)**	**0.27 (0.08–0.91)**	0.44 (0.09–2.06)	0.52 (0.13–2.18)	0.64 (0.19–2.12)	0.65 (0.20–2.05)

Ref ,  Reference group; OR , Odds ratio; aOR,  Adjusted odds ratio. Statistically significant findings are bolded. For contraceptive use at first sexual intercourse, adjusted models controlled for age at first sex, poverty level, childhood family structure, religious affiliation, and relationship with first male sexual partner. For contraceptive use at most recent intercourse, adjusted models controlled for age at interview, poverty level, childhood family structure, religious affiliation, relationship with last male sexual partner, duration of past-year health insurance coverage, and parity

## Discussion

The goals of this study were to identify and describe patterns of parental sexual socialization messages in a nationally representative sample of young Black women and adolescent girls, and examine the associations between the socialization patterns and contraceptive behaviors at the first and most recent acts of sexual intercourse. We identified four patterns of parental sexual socialization content, which differed significantly in sociodemographics, age at first sex, number of socialization messages received, and contraceptive behaviors, particularly at the first intercourse. These findings elucidate the notable heterogeneity in sexual socialization experiences of young Black women and adolescent girls in the United States. Moreover, the findings have important practice implications for identifying young Black women and girls in the U.S. population who may be at greater risk of unintended pregnancies, and for providing guidance to Black parents as they socialize Black girls about sex and related topics. This is particularly salient given the current state of reproductive rights in the U.S., including restrictive abortion policies across many states (Guttmacher Institute, 2024).

The identification of a *Comprehensive Socialization* class and a *Limited Socialization* class in this sample is consistent with the broader literature that some Black girls receive comprehensive and instructive sexual socialization messages from their families, while others receive little to no socialization (Brown et al., 2024; Leath et al., 2020). The *Abstinence-focused Socialization* class also corresponds with previous research that Black girls are often socialized to abstain from sexual activities, and these messages often contain warnings about the adverse consequences of sex, including STDs (Dennis & Wood, 2012; Frederick et al., 2024). In contrast, the finding that some Black women and girls receive *Contraception-focused Socialization* is novel, though this pattern represented only 10% of the sample. While some studies show that Black girls may receive messages about contraception in their socialization (DiIorio et al., 1996), others have found that some parents discourage or avoid talking about contraception with Black girls (Logan et al., 2021). Furthermore, this class was characterized by a low probability of receiving messages about how to use a condom, which could also reflect parents’ unwillingness to discuss or discomfort in discussing how to properly use condoms with their daughters (Pluhar & Kuriloff, 2004). It could also be that some parents hold unfavorable views of condoms as an effective method of contraception, in comparison to other methods. Research shows that parents of adolescents hold more favorable views of more effective forms of contraception such as oral contraceptive pills than of condoms (Eisenberg et al., 2004; Hartman et al., 2013).

In addition to identifying sexual socialization patterns, we found that respondents in the *Limited Socialization* class were younger at the time of their first sexual intercourse in comparison to those in the *Abstinence-focused* and *Comprehensive Socialization* classes. This adds to the mixed body of research on the relationship between sexual socialization and age at sexual debut that has been reported in the general population (Coakley et al., 2017; DiIorio et al., 2003), and in samples of Black women and girls (Bynum, 2007; Donenberg et al., 2011; Usher-Seriki et al., 2008). Nonetheless, these findings suggest that minimal parental engagement in sexual socialization may not promote delayed sexual activity in adolescents. We also found that only a small proportion of religiously affiliated respondents were classified in the *Contraception-focused* class, in comparison to those without a religious affiliation. In contrast, a larger proportion of religiously affiliated respondents were classified in the *Abstinence-focused Socialization* class than those without a religious affiliation. For some religions such as Catholicism and specific Protestant groups, there are specific guidelines that either prohibit the use of contraception or specific contraceptive methods, and instead favor abstinence as an acceptable form of contraception (Srikanthan & Reid, 2008). Thus, sexual socialization practices that primarily center contraception may be uncommon among some religious families, especially those with religious beliefs that oppose contraception.

Furthermore, we found that respondents in the *Comprehensive Socialization* class received more socialization messages than those in the other three patterns, whereas respondents in the *Limited Socialization* class received the lowest number of messages of the four patterns. Notably, respondents in the *Abstinence-focused* and *Contraception-focused* classes received the same number of socialization messages, but they differed in the types of messages they received, which is what we aimed to capture in our latent class analysis. Had we simply summed the number of socialization messages received as most extant research in this area does, those in the *Abstinence-focused* class would be similarly categorized as those in the *Contraception-focused* class. Consequently, these findings emphasize that summing across indicators of sexual socialization to assess socialization experiences does not effectively capture differences in the content of sexual socialization messages, which may have differential associations with sexual and reproductive health outcomes.

Moreover, we found that compared to respondents in the *Contraception-focused Socialization* class, those in the *Limited Socialization* and *Abstinence-focused Socialization* classes were less likely to have used any form of contraception, long- or short-acting reversible contraceptive methods, or multiple contraceptive methods at the first sexual intercourse. These findings may be explained in part by the considerable differences between the socialization groups in the receipt of messages about the different types of contraceptive methods and where to access them. Respondents in the *Contraception-focused* class may be more informed about contraceptive methods and their effectiveness than those in the other two groups, and therefore engage in more preventive behaviors. Furthermore, the use of multiple methods concurrently by respondents in the *Contraception-focused* class may be due to heightened vigilance about preventing pregnancies, given their socialization experiences. This is consistent with prior research that has found that reproductive-aged women use multiple methods simultaneously due to fears of experiencing an unintended pregnancy (Frohwirth et al., 2016). In contrast, there were no significant differences in contraceptive practices at the first intercourse between respondents in the *Contraception-focused* and *Comprehensive Socialization* classes, but these two groups had similar probabilities of having received contraceptive-specific messages.

We found few statistically significant differences in the contraceptive outcomes assessed at the most recent sexual intercourse by the patterns of sexual socialization, particularly between the *Limited Socialization* and *Contraception-focused* classes. Respondents in the *Limited Socialization* class were less likely to have used any form of contraception or multiple methods at the most recent sex, compared to those in the *Contraception-focused* class. Furthermore, results from sensitivity analyses showed that in the unadjusted model, respondents in the *Limited Socialization* class were less likely to have used a long- or short-acting reversible method versus no method than those in the *Contraception-focused* class. These findings suggest that parental sexual socialization messages may be somewhat less pertinent to contraceptive use at later acts of intercourse as compared to the first act, though meta-analytic evidence suggests that parental influence may persist over time in adolescents and young adults (Widman et al., 2016). Nonetheless, it is possible that as Black girls become more exposed to other sources of influence such as peers or the media, their parents’ influence on their sexual attitudes and behaviors may weaken over time. However, these findings should be interpreted with caution since we did find strong effects, albeit non-significant, that were in the same direction as our findings on contraceptive use at first sex as evidenced in Tables 3, 4 and 5 and Supplementary Table 3. This suggests that the study may have lacked statistical power to reliably detect these effects.

### Limitations and future research directions

There are some limitations of this study that are important to consider. First, the items on sexual socialization only assessed whether respondents received messages about specific topics, but did not assess the frequency or quality of the messages, or the timing of parental sexual socialization (e.g., before or after first sexual intercourse). Future research should utilize sexual socialization measures that assess the frequency, content, quality, and timing of young Black women and girls’ socialization experiences. Second, the reports of socialization experiences may have been subject to recall bias, and the cross-sectional design of the NSFG limits inferences about causality of the associations. Third, only a few respondents (2%) reported using long-acting reversible methods at the first intercourse, which prevented us from testing whether the sexual socialization patterns differed in the use of these methods at the first intercourse. Finally, compared to the full sample size, the sample size for analyses examining the associations between the socialization patterns and contraceptive practices at the most recent intercourse was smaller, and may have limited our ability to detect effects. Additional research is warranted to investigate the role of parental sexual socialization experiences on later acts of sex in young, sexually active Black women and adolescent girls.

Taken together, this study helps to inform future research on parental sexual socialization and sexual and reproductive health in young Black women and adolescent girls. Future studies should investigate whether parental sexual socialization experiences are associated with other sexual and reproductive health attitudes, behaviors and outcomes such as pregnancy desire and pregnancy occurrences. In addition, longitudinal research is needed to establish the temporal ordering of parental sexual socialization and sexual and reproductive health attitudes, behaviors and outcomes in young Black women and girls. Longitudinal research should also be conducted to identify potential psychological and sociocultural mechanisms through which parental sexual socialization experiences are linked to sexual and reproductive health in young Black women and adolescent girls.

### Practice implications

The findings from this study highlight that there is no singular approach to parental sexual socialization of Black girls in the United States. Furthermore, our study underscores the influential role of Black families on the sexual and reproductive health experiences of Black girls. Specifically, young Black women and adolescent girls who received comprehensive sexual socialization or contraception-focused socialization from their parents were more likely to engage in practices to prevent pregnancies than those whose parents primarily endorsed abstinence or engaged in little to no sexual socialization. These findings suggest that parental sexual socialization that includes messages about contraception may reduce the risk of adverse sexual and reproductive health experiences such as contraceptive nonuse and unintended pregnancies in young Black women and adolescent girls. Thus, it is crucial that Black parents engage in discussions about contraception with their daughters, specifically about the various types of contraceptive methods and how to access them.

The findings also have important implications for educators, clinicians, and researchers in supporting Black parents in their sexual socialization of Black girls. Research shows that parents may avoid discussing sex with their children due to discomfort, embarrassment, and lack of knowledge (DiIorio et al., 2003; Flores & Barroso, 2017). Thus, educators and clinicians can assist Black parents in their sexual socialization by providing them with informative sexual health resources, while also being cognizant of historical, cultural, and familial factors that may influence how they talk about sex with their daughters. In addition, clinicians and educators should consider the parental sexual socialization experiences of young Black women and adolescent girls when engaging with them, and tailor their services or educational messages to meet their unique needs. Finally, the findings underscore the need for culturally relevant, family-based interventions to assist Black parents in expanding their knowledge of contraception, and supporting parent-child discussions about contraception.

## Conclusion

In summary, we identified four patterns of parental sexual socialization in a nationally representative sample of young Black women and adolescent girls, which underscore differences in the sexual socialization experiences of young Black women and adolescent girls in the United States. In addition, compared to Black women and girls who received contraception-focused socialization messages, those who received limited sexual socialization or abstinence-focused messages were less likely to engage in preventive contraceptive behaviors at the first sexual intercourse. However, there were no differences between Black women and girls who received contraception-focused messages versus comprehensive sexual socialization messages in behaviors at the first intercourse. Furthermore, there were few differences between the socialization patterns in contraceptive practices at the most recent intercourse. This study adds to the limited body of research examining the association between parental sexual socialization and contraceptive use in young Black women and girls, and may inform future research on the role of parental sexual socialization on attitudes and behaviors concerning contraception in this population.

## Data Availability

The data are publicly available and the analytic scripts are available upon request.
